# Engineering of Genetically Arrested Parasites (GAPs) For a Precision Malaria Vaccine

**DOI:** 10.3389/fcimb.2017.00198

**Published:** 2017-05-31

**Authors:** Oriana Kreutzfeld, Katja Müller, Kai Matuschewski

**Affiliations:** Department of Molecular Parasitology, Institute of Biology, Humboldt UniversityBerlin, Germany

**Keywords:** malaria, *Plasmodium*, vaccine, liver stage, live attenuated parasite, sporozoite, immune memory

## Abstract

Continuous stage conversion and swift changes in the antigenic repertoire in response to acquired immunity are hallmarks of complex eukaryotic pathogens, including *Plasmodium* species, the causative agents of malaria. Efficient elimination of *Plasmodium* liver stages prior to blood infection is one of the most promising malaria vaccine strategies. Here, we describe different genetically arrested parasites (GAPs) that have been engineered in *Plasmodium berghei, P. yoelii* and *P. falciparum* and compare their vaccine potential. A better understanding of the immunological mechanisms of prime and boost by arrested sporozoites and experimental strategies to enhance vaccine efficacy by further engineering existing GAPs into a more immunogenic form hold promise for continuous improvements of GAP-based vaccines. A critical hurdle for vaccines that elicit long-lasting protection against malaria, such as GAPs, is safety and efficacy in vulnerable populations. Vaccine research should focus on solutions toward turning malaria into a vaccine-preventable disease, which would offer an exciting new path of malaria control.

## Introduction

Malaria remains the most important vector-borne infectious disease and affects half of the world's population. Globally, >200 million infected individuals develop clinical symptoms, and >400,000 die because of severe malaria, primarily children in Sub-Saharan Africa (WHO, 2016a). New strategies for malaria prevention and eradication are thus urgently required. The current malaria control programs target the causative agents, *Plasmodium falciparum, P. vivax*, and three other human-infecting *Plasmodium* parasites, at different life cycle stages, which together reduces morbidity and mortality in endemic regions. Attacking the parasite at its vector stage with long-lasting insecticide treated bed nets and insecticide-indoor residual spraying combined with access to rapid diagnosis and artemisinin-based combination therapy for clinical malaria episodes are recommended by the WHO (WHO, 2016a).

Repeated exposure to *Plasmodium* transmission in malaria-endemic countries leads only to very slow acquisition of naturally acquired immunity that rapidly wanes. Development of protective immunity is likely hindered by blood infection, the exclusive cause of malaria-related morbidity and mortality. Therefore, the pre-erythrocytic phase of the *Plasmodium* life cycle in the mammalian host is particularly attractive as immunization agent since no clinical symptoms are associated with this first replication phase (Prudêncio et al., 2006; Silvie et al., 2008a; Matuschewski et al., 2011). It also allows priming of CD4^+^ and CD8^+^ T cells by presenting parasite antigens to the host immune system *via* MHCI and MHCII by dendritic cells and MHCI by infected hepatocytes, respectively (Hafalla et al., 2011). Prevention of disease by vaccination is an ideal addition to the portfolio of malaria intervention tools; however, it remains one of the greatest challenges in medical research.

Approaches to design a protective long-lasting malaria vaccine are wide-ranging, and some of them have already reached clinical trial phases (WHO, 2016b). Among these candidates is RTS,S/AS01, which is the first malaria vaccine that achieved licensure for administration in malaria endemic areas. RTS,S/AS01 is a hepatitis B-based subunit vaccine and contains a fragment of the circumsporozoite protein (CSP) (Stoute et al., 1997). Recent phase III clinical trial results revealed an unfavorable protection level casting doubt on the impact of RTS,S/AS01 in malaria control efforts (RTS,S Clinical Trials Partnership, 2011; Olotu et al., 2016).

A benchmark for a malaria vaccine exists for several decades already, and this experimental vaccine with proven long-term protection is a whole sporozoite vaccine. Small immunization studies in mice, non-human primates, and humans demonstrated that radiation attenuated sporozoites (RAS) elicit sterile protection against *Plasmodium* challenge infections (Nussenzweig et al., 1967, 1969; Clyde et al., 1973; Gwadz et al., 1979; Hoffman et al., 2002). Irradiation induces DNA breakage in the parasites, which reduces the numbers of nuclear divisions and limits liver stage expansion to early schizonts (Silvie et al., 2002). Timing and radiation dosage is critical since over-irradiated sporozoites arrest early at the unicellular stage, thus decreasing protection in experimental cohorts (Friesen and Matuschewski, 2011). Successful hepatocyte invasion and initial intra-hepatic development of live, metabolically active sporozoites are required to elicit strong immunity, and, therefore, pose a substantial hurdle toward an affordable and undemanding sporozoite vaccine. The central importance of live and metabolically active sporozoites has been corroborated with heat-killed sporozoites, which elicit only very weak and short-term, antibody-mediated protection against subsequent *Plasmodium* sporozoite challenge infections (Hafalla et al., 2006). On the other hand, suboptimal irradiation harbors the risk of breakthrough infections during vaccination—a considerable drawback concerning safety. RAS arrest early during liver stage development and express primarily sporozoite-derived antigens, including CSP and thrombospondin-related anonymous protein (TRAP), which likely contribute to priming of T cell-mediated immunity.

Liver stage developmental arrest can also be achieved by an alternative approach, termed chemical attenuation of sporozoites (CAS), which is based on simultaneous administration of normal sporozoites and anti-malarial drugs (Belnou et al., 2004; Putrianti et al., 2009; Roestenberg et al., 2009; Friesen et al., 2010; Bijker et al., 2013). While they offer interesting evaluations in small-scale exploratory clinical studies, these approaches are critically reliant on continuous clinical supervision during drug administration and currently bear no translational perspective.

The potential to induce lasting protection by live attenuated, metabolically active parasites led to the engineering of genetically attenuated parasites (GAPs) as tailored whole parasite vaccines against malaria infections (Mueller et al., 2005a). Murine malaria models employing the rodent malaria parasites *P. berghei* and *P. yoelii* enable the exploration of liver stage-specific proteins and their importance for parasite survival. In the past years, over 120 genes have been targeted by experimental genetics and analyzed for defects during the *Plasmodium* life cycle in both vector and mammalian host (Janse et al., 2011). Many gene knockouts resulted in normal parasite life cycle progression or refractoriness to targeted deletion, indicative of redundant functions during life cycle progression or vital roles for blood infection, respectively. Additionally, arrest of the parasite development in the mosquito midgut, prior to salivary gland colonization, or ahead of hepatocyte invasion was frequently observed. Accordingly, only very few candidate genes fulfill the criteria of potential GAP vaccine candidate lines (Table 1).

**Table 1:** *****Plasmodium*** genes targeted for GAPs. ***Plasmodium berghei*** (black), ***P. yoelii*** (green) and ***P. falciparum*** (blue) are listed**.

**Target gene**	**Gene IDs**	**Liver stage arrest**	**Breakthroughs**	**References^a^**
**ETRAMPs/PV PROTEINS**				
*UIS3*	PBANKA_1400800	Early	No	Mueller et al., 2005a
	PY17X_1402400	Early	No	Tarun et al., 2007
*UIS4*	PBANKA_0501200	Early	Yes	Mueller et al., 2005b
	PY17X_0502200	Early	No	Tarun et al., 2007
**6-CYS PROTEINS**				
*P36, P36p (P52)*	PBANKA_1002100,	Early	Yes	van Dijk et al., 2005,
	PBANKA_1002200	Early		Ishino et al., 2005
	PY17X_1003500,	Early	No	Labaied et al., 2007
	PY17X_1003600	Early		
	PF3D7_0404400,	Early	Yes	van Schaijk et al., 2008
	PF3D7_0404500	Early		
*B9*	PBANKA_0808100	Early	Yes	Annoura et al., 2014
	PF3D7_0317100	Early	Yes	van Schaijk et al., 2014a
**DIFFERENTIATION FACTORS**				
*SLARP (SAP1)*	PBANKA_ 090210	Early	No	Silvie et al., 2008b
	PY17X_0903500	Early	No	Aly et al., 2008
	PF3D7_1147000	Early	No	van Schaijk et al., 2014a
**METABOLISM**				
*FabI*	PBANKA_1229800	Late	Yes	Yu et al., 2008
*FabB/F*	PBANKA_1125100	Late	Yes	Annoura et al., 2012
	PY17X_1126500	Late	No	Vaughan et al., 2009
*FabZ*	PY17X_1342900	Late	No	Vaughan et al., 2009
*G3PAT*	PBANKA_1416700	Late	Yes	Shears et al., 2017
	PY17X_1418400	Late	No	Lindner et al., 2014
*G3PDH*	PY17X_0934900	Late	No	Lindner et al., 2014
*PDH-E1a*	PBANKA_0923800	Late	Yes	Nagel et al., 2013
	PY17X_0925800	Late	No	Pei et al., 2010
*PDH-E3*	PY17X_0715100	Late	No	Pei et al., 2010
**TRANSPORTERS**				
*MFS6*	PBANKA_1304700	Late	Yes	Kenthirapalan et al., 2016
**FUNCTION UNKNOWN**				
LISP2	PBANKA_1003000	Late	Yes	Orito et al., 2013
*PALM*	PBANKA_0101100	Very late	Yes	Haussig et al., 2011
*PlasMei2*	PY17X_1123700	Late	No	Dankwa et al., 2016
*SPELD*	PBANKA_0910900	Mid	Yes	Al-Nihmi et al., 2017
PKG^b^	PBANKA_1008200	Late	No	Falae et al., 2010

a *References list only the first report in the respective Plasmodium species*.

b *Incomplete stage-specific gene knockout*.

Herein, we review the most recent developments in GAP vaccine discovery. We assess the different GAPs that have been generated in the last 12 years and evaluate their vaccine potential. Finally, we address the challenges and obstacles in designing a GAP vaccine for vulnerable populations in malaria-endemic countries.

## First generation GAPs: proof of principle studies

**Figure 1:**
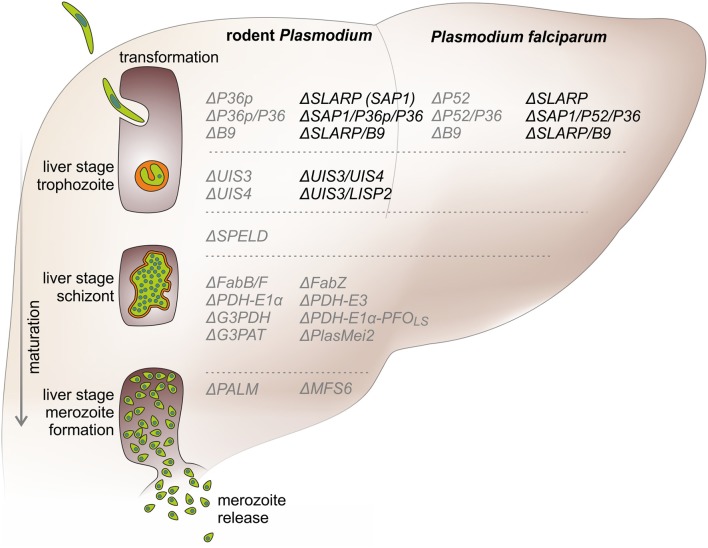
**Precision developmental arrests during ***Plasmodium*** liver stage maturation by genetically arrested parasites (GAPs)**. A sporozoite (green) invades a suitable hepatocyte under simultaneous formation of a replication-competent intracellular organelle, termed parasitophorous vacuole (orange). After intracellular transformation to round early liver stages, the parasite expands to a single-cell liver stage trophozoite. Early arresting GAPs, *e.g*., Δ*UIS3*, Δ*SLARP*, Δ*P36p*, stop cell division at this stage. Next, the trophozoite grows in size and replicates and eventually forms a schizont that exceeds the size of the original host cell. Many GAP lines, such as deletions of the FASII biosynthesis pathway or other apicoplast functions, *e.g*., Δ*PALM*, arrest after full liver stage maturation. In the last phase of pre-erythrocytic development, the first generation of erythrocyte-invading stages, termed merozoites are formed and released from the infected hepatocytes into the blood stream. This step marks the transition from the clinically and diagnostically silent liver phase to the blood infection, which is responsible for all malaria-related symptoms and pathology. GAPs (right) are listed next to the phase of liver stage development (left) according to their life cycle arrest. Safe and unsafe GAPs are depicted in black and gray, respectively. Knockouts of the murine and human *Plasmodium* species are shown on the left and right side, respectively.

The first preclinical studies that showed successful generation of GAP lines and their efficacy in immunization protocols targeted *Plasmodium berghei* genes that represent members of the early transcribed membrane protein (ETRAMP) family (Mueller et al., 2005a,b). The selected genes, i.e., upregulated in infective sporozoites gene 3 (*UIS3*) and *UIS4*, fulfilled three principal criteria: (i) stage-specific gene expression in pre-erythrocytic stages, thereby allowing recombinant knockout strains to be selected during blood stage transfection; (ii) abundant expression in pre-erythrocytic stages, indicating likely vital roles during this phase of the life cycle; and (iii) genes that are unique to *Plasmodium* and related haemosporidian parasites, which all share the hallmark of the first obligate population expansion phase in the host liver (Matuschewski et al., 2002). *In vitro* studies in cultured hepatoma cells revealed that *P. berghei* as well as *P. yoelii* Δ*UIS3* and Δ*UIS4* parasite lines arrest early in liver stage development after completion of sporozoite transformation to liver stages, but before onset of parasite replication (Figure 1; Mueller et al., 2005a,b; Tarun et al., 2007).

Immunization studies with one prime and two booster vaccinations showed complete long-term protection with *P. berghei* and *P. yoelii* Δ*UIS3* and Δ*UIS4* GAPs in C57BL/6 and BALB/cJ mice, respectively (Table 2) (Mueller et al., 2005a,b; Tarun et al., 2007). The study on *P. berghei* Δ*UIS4* GAPs established frequent occurrence of breakthrough infections, *i.e*., a proportion of animals develop blood infections during the immunization procedure (Mueller et al., 2005b). This safety concern needs careful examination before progression of *P. falciparum* GAPs to human testing can take place (Matuschewski, 2013). Notably, the identification of orthologous genes for *UIS3* and *UIS4* in *P. falciparum* has so far remained elusive, although various ETRAMPs emerge as potential candidates (Spielmann et al., 2012). To date, only one *P. falciparum ETRAMP* has been targeted by reverse genetics (MacKellar et al., 2010). This protein, termed *Pf* ETRAMP 10.3, apparently performs essential functions during blood infection, since it remains refractory to targeted deletion and does not complement the *P. yoelii* Δ*UIS4* defects. Accordingly, more research is needed to identify and target *P. falciparum* ETRAMPs to generate this class of GAPs in *P. falciparum* and test their vaccine potential in small-scale human trials.

**Table 2:** *****P. berghei*** and ***P. yoelii*** GAPs: long-term immunization studies**.

**GAPs^a^**	**Immunization dose (days after priming)**			**Challenge^b^ (days after last boost)**	**No. protected/No. challenged animals^c^**	**References**
	** 1**	**2**	**3**			
**ETRAMPS**						
Δ*UIS3*	50 k	25 k (34)	25 k (45)	10 k (30)	5/5 (100%)	Mueller et al., 2005a
	10 k	10 k (34)	10 k (45)	10 k (30)	5/5 (100%)	
	10 k	10 k (14)	10 k (28)	10 k (60)	4/4 (100%)	Tarun et al., 2007
				(180)	8/12 (67%)	
	10 k	10 k (14)	−	10 k (30)	4/4 (100%)	
Δ*UIS4*	50 k	25 k (14)	25 k (28)	50 k (38)	8/8 (100%)	Mueller et al., 2005b
	10 k	10 k (14)	10 k (28)	50 k (38)	8/8 (100%)	
	10 k	10 k (14)	10 k (28)	10 k (60)	4/4 (100%)	Tarun et al., 2007
				(180)	8/8 (100%)	
	50 k	−	−	10 k (30)	4/4 (100%)	
Δ*UIS3/UIS4*	75 k	20 k (7)	20 k (14)	10 k (180)	6/6 (100%)	Jobe et al., 2007
	10 k	10 k (14)	10 k (28)	10 k (118)	14/14 (100%)	
Δ*UIS3/LISP2*	50 k	20 k (14)	20 k (28)	10 k (42)	8/8 (100%)	Kumar et al., 2016
				(102)	8/8 (100%)	
**6-CYS PROTEINS**						
Δ*P36p*	50 k	20 k (7)	20 k (14)	10 k (30)	5/5 (100%)	van Dijk et al., 2005
Δ*P52/P36*	10 k	10 k (7)	10 k (14)	10 k (30)	7/7 (100%)	Labaied et al., 2007
Δ*B9*	50 k	20 k (7)	20 k (14)	10 k (90)	5/5 (100%)	van Schaijk et al., 2014a
				(180)	9/9 (100%)	
				(365)	5/11 (45%)	
**DIFFERENTIATION FACTORS**						
Δ*SLARP* (ΔSAP1)	50 k	25 k (14)	25 k (28)	10 k (42)	5/5 (100%)	Silvie et al., 2008b
				(98)	2/5 (40%)	
	10 k	10 k (14)	10 k (28)	10 k (36)	1/4 (25%)	
	1 k	1 k (14)	1 k (28)	10 k (36)	0/4 (0%)	
	10 k	10 k (14)	10 k (28)	10 k (30)	25/25 (100%)	Aly et al., 2008
				10 k (120)	25/25 (100%)	
	1 k	1 k (14)	1 k (28)	10 k (30)	10/10 (100%)	Butler et al., 2011
	1 k	1 k (14)	−	10 k (30)	2/10 (20%)	
Δ*SLARP/B9*	50 k	25 k (7)	25 k (14)	10 k (180)	6/6 (100%)	van Schaijk et al., 2014a
Δ*SAP1/P52/P36*	10 k	10 k (14)	−	10 k (30)	5/5 (100%)	Kublin et al., 2017
				(180)	5/5 (100%)	
**FATTY ACID SYNTHESIS PATHWAY**						
Δ*FabB/F*	50 k	50 k (14)	50 k (28)	10 k (210)	8/8 (100%)	Butler et al., 2011
	10 k	10 k (14)	10 k (28)	10 k (100)	5/5 (100%)	
				(300)	8/8 (100%)	
	10 k	10 k (14)	10 k (28)	10 k (30)	8/8 (100%)	
	1 k	1 k (14)	1 k (28)	10 k (30)	10/10 (100%)	
	1 k	1 k (14)	−	10 k (30)	10/10 (100%)	
Δ*G3PAT*	10 k	10 k (37)	−	10 k (37)	30/30 (100%)	Lindner et al., 2014
Δ*G3PDH*	10 k	10 k (37)	−	10 k (37)	30/30 (100%)	Lindner et al., 2014
Δ*PDH-E1*-*PFO_*LS*_*	5 k	5 k (25)	5 k (50)	5 k (30)	10/10 (100%)	Nagel et al., 2013
**OTHER FUNCTIONS**						
Δ*PALM*	10 k	10 k (~30)	−	10 k (~30)	5/5 (100%)	Haussig et al., 2011
				(110)	6/7 (86%)	
Δ*MFS6*	10 k	10 k (9)	−	10 k (22)	2/2 (100%)	Kenthirapalan et al., 2016
	1 k	1 k (9)	−	10 k (22)	1/12 (8%)	
Δ*PlasMei2*	10 k	10 k (35)	10 k (45)	10 k (~40)	21/21 (100%)	Dankwa et al., 2016

a *P. berghei and P. yoelii knockout parasite lines are displayed in black and gray, respectively*.

b *Only studies where i. v. challenge was performed at least 3 weeks after the last boost are listed*.

c *Immunizations were performed in the P. berghei-C57BL/6 and P. yoelii-BALB/cJ combinations, respectively*.

Another first generation GAP line in *P. berghei* and *P. yoelii* are parasites that lack *P36p* (also termed *P52*), a member of the *Plasmodium*-specific 6-Cys protein family (van Dijk et al., 2005). Parasites lacking *P36p* (*P52*) or its paralog, *P36*, arrest again early during liver stage development after completion of sporozoite transformation (Figure 1) (Ishino et al., 2005; van Dijk et al., 2005). Δ*P36p* sporozoites transmigrate and invade hepatocytes, wherein they initiate the formation of a parasitophorous vacuole membrane (PVM). Although, they start to mature into liver stage trophozoites, Δ*P36p* parasites suddenly abort this development, most likely because maintenance and maturation of the PVM are critically impaired (van Dijk et al., 2005).

Once more, occasional breakthrough infections in mice inoculated with Δ*P36p* sporozoites were observed (van Dijk et al., 2005). Interestingly, in the Δ*P36p*/*P36* double knockout parasite expression of the signature merozoite surface protein 1 (MSP1) could not be detected, and the mechanism of breakthrough infections remains unsolved (Ploemen et al., 2012). Targeted gene deletion of a second member of the 6-Cys family, termed *B9*, led to similar early arrest and occasional breakthrough infections (Annoura et al., 2014).

Since genes of the 6-Cys protein family are remarkably conserved across *Plasmodium* species, *P. falciparum* GAP lines were generated to show proof of principle of liver stage attenuation by targeted gene deletion in human malarial parasites (van Schaijk et al., 2008; van Buskirk et al., 2009). However, in order to advance this approach to clinical testing in humans, safety is of utmost importance. Accordingly, it remains enigmatic why a *Pf* GAP based on *P52* (*P36p*) and *P36* was selected for a first phaseI/IIa clinical trial (Spring et al., 2013), despite the alarming preclinical data. As predicted, human trials with PfΔ*P52/P36* had to be suspended because of breakthrough infections (Spring et al., 2013).

A potential, albeit untested, advantage of 6-Cys protein-based GAPs is enhanced antigen presentation via MHCI on infected hepatocytes, since maintenance of the parasitophorous vacuole is impaired (van Dijk et al., 2005). Perhaps even more important, the propensity to increase apoptosis in Δ*P36p*-infected hepatocytes (van Dijk et al., 2005) might enhance cross-priming by dendritic cells (DCs) that phagocytose apoptotic bodies (Leiriao et al., 2005), although this conjecture remains controversial (Renia et al., 2006).

Together, the first generation GAPs established that precise developmental arrests during the first clinically silent, intrahepatic *Plasmodium* expansion phase can be engineered by tailored removal of individual vital genes from the entire *Plasmodium* genome (Table 1). These uniform, genetically defined parasites consistently elicit lasting protection against sporozoite challenge infections in vaccination protocols with three consecutive GAP sporozoite inoculations (Table 2).

## GAPs targeting liver stage differentiation: safety first

The observations of breakthrough blood infections during the immunization protocol in a proportion of animals (Mueller et al., 2005b; van Dijk et al., 2005) initiated the search for candidate *Plasmodium* genes that are key developmental factors, for instance transcription factors at the nexus of sporozoite to liver stage transformation. Such a factor was identified during the analysis of sporozoite-specific (S) genes (Kaiser et al., 2004). Targeted gene deletion of this factor, termed S22 or sporozoite and liver stage asparagine-rich protein (SLARP or SAP1) (Aly et al., 2008; Silvie et al., 2008b), resulted in a complete arrest of the parasite at early liver stage development prior to nuclear division (Figure 1). A novel hallmark of Δ*SLARP* parasites was the differential down-regulation of many liver stage-specific mRNAs and their corresponding proteins, including PVM-resident proteins such as *UIS3* and *UIS4* (Aly et al., 2008; Silvie et al., 2008b), suggesting pleiotropic defects as a result of the absence of a major transcriptional regulator of liver stage differentiation.

Most importantly, Δ*SLARP* parasites are the first and only GAPs reported to fulfill all criteria of safe arrest (Aly et al., 2008; Silvie et al., 2008b). Accordingly, after the safety failure of PfΔ*P52/P36*, all *Pf* GAPs developed for clinical testing in humans include the corresponding *PfSLARP* knockout (Mikolajczak et al., 2014; van Schaijk et al., 2014a; Kublin et al., 2017). However, immunization studies showed a lack in long-term protection in animals immunized with *P. berghei* Δ*SLARP* parasites, where only 40% of all animals were protected 3.5 months after immunization (Table 2) (Silvie et al., 2008b). It is conceivable that Δ*SLARP* parasites display a smaller array of antigens, but this has to be experimentally tested employing systems immunology approaches.

Notably, targeting of other key factors important for liver stage gene expression did not result in a similar complete liver stage arrest. Deletion of the liver stage-specific transcription factor of the apetala 2 family, termed *AP2-L*, only resulted in a developmental delay (Iwanaga et al., 2012). Knockout of the eukaryotic initiation factor 2α (eIF2α) kinase (*IK2/UIS1*), which is critical for sporozoite latency, and stage-specific knockout of the corresponding eIF2α-P protein phosphatase 1 (*PP1/UIS2*), which is a regulator of protein translation after hepatocyte invasion, led to incomplete early arrests before and immediately after hepatocyte invasion (Zhang et al., 2010, 2016). Targeted deletion of another regulator of sporozoite latency, the RNA-binding protein *PUF2*, reproduced the Δ*IK2/UIS1* phenotype, again with an unsatisfactory safety profile (Gomes-Santos et al., 2011; Müller et al., 2011).

In conclusion, immunization data together with the demonstration of a very early, complete arrest indicate that Δ*SLARP* parasites are comparable to RAS, with the important distinctions of a precision life cycle arrest in humans (Kublin et al., 2017) and safe handling of Δ*SLARP*-infected *Anopheles* mosquitoes for vaccine production.

## Late arresting GAPs: improved immunogenicity but lack of safety

Studies employing co-administration of normal sporozoites and anti-malarial drugs have consistently shown superior immunity of late liver stage and/or early blood cycle arrest in murine malaria models (Belnou et al., 2004; Friesen et al., 2010; Friesen and Matuschewski, 2011) and small scale human trials (Roestenberg et al., 2009; Bijker et al., 2013), suggesting that a late liver stage arrest offers multiple advantages, perhaps including broader antigen presentation (Borrmann and Matuschewski, 2011). Unexpectedly, antibiotic-induced arrest at the transition from late liver stages to blood infection leads to better protection than a later arrest, after a few rounds of blood stage replication, induced by chloroquine treatment (Friesen and Matuschewski, 2011). This indicates an immune-modulatory effect by infected red blood cells and potential benefits of a complete arrest at the liver stage.

Therefore, tailored arrest toward the end of liver stage maturation was an important third step in GAP vaccine design. A CAS-based arrest using the antibiotic azithromycin showed that specific targeting of the *Plasmodium* apicoplast, a relict non-photosynthetic plastid organelle, resulted in late arrest after complete liver stage maturation (Friesen et al., 2010). Accordingly, two complementary approaches targeting key factors in the *Plasmodium* apicoplast led to generation of late-arrested GAPs and their testing in vaccine studies (Figure 1), namely deletion of a fatty acid biosynthesis enzyme (Butler et al., 2011) and a *Plasmodium*-specific protein of unknown function (Haussig et al., 2011). In both cases potent protection against reinfection was reported and superior protection correlated with extended liver stage maturation.

The fatty acid synthesis II (FASII) pathway produces saturated fatty acids in the apicoplast. It includes a cyclic reaction that catalyzes fatty acid elongation and a large pyruvate dehydrogenase (PDH) complex that forms acetyl-CoA for the elongation cycles (Yu et al., 2008). Targeted deletion of an enzyme of the cyclic reaction, namely *trans*-2-enoyl-ACP reductase (*FabI*), in *P. berghei* revealed a specific defect during liver stage maturation (Yu et al., 2008). However, the life cycle arrest was incomplete and sporozoite inoculations resulted in substantial breakthrough infections in C57BL/6 mice (Table 1) (Yu et al., 2008). Accordingly, immunization studies were not performed.

The liver stage defects of mutants in the FASII biosynthesis pathway in murine malaria parasites were confirmed in *P. yoelii* studies by targeted deletion of 3-oxoacyl-ACP synthase I/II (*FabB/F*) and β-hydroxyacyl-ACP dehydratase (*FabZ*) (Vaughan et al., 2009). Since breakthrough infections were once more absent in the *P. yoelii* model testing of Δ*FabB/F* parasites as late-arrested GAPs in vaccination protocols was possible (Table 2) (Butler et al., 2011). This study reported better protection and a larger CD8^+^ T cell response in comparison to Δ*SAP1* and RAS parasites. Targeted gene deletion of additional *P. yoelii* enzymes of the FASII and the subsequent lipid biosynthesis pathway resulted in similar arrests in liver schizont maturation prior to merozoite formation (Pei et al., 2010; Lindner et al., 2014). The target genes were the E1α and E3 subunits of PDH, glycerol-3-phosphate acyltransferase (G3PAT), and glycerol-3-phosphate dehydrogenase (G3PDH) (Figure 1) of which the latter performed well in vaccine protocols (Table 2).

The previous observation of breakthrough infections in the first study of a *P. berghei* FASII pathway knockout (Yu et al., 2008) was subsequently confirmed (Table 1) (Annoura et al., 2012; Shears et al., 2017) and strictly limits the results from studies in *P. yoelii*. The additional, unexpected finding of aborted parasite development in mosquitos infected with the corresponding *P. falciparum* knockouts (Cobbold et al., 2013; van Schaijk et al., 2014b) essentially eliminated the possibility to develop GAP vaccines by targeted deletion of the FASII pathway. Together, these results also raise the important question, which prerequisites have to be fulfilled to transfer discoveries from murine models to *Pf* GAP vaccines.

The second strategy of targeting essential apicoplast functions to generate late liver stage-arrested parasite lines built upon bioinformatic prediction of *Plasmodium*-specific apicoplast targeted proteins (Haussig et al., 2011). The first target that satisfied these criteria was *P. berghei Plasmodium*-specific apicoplast protein important for liver merozoite formation (PALM), a protein of unknown function. Targeted deletion of *PALM* did not affect parasite growth or apicoplast morphology, but resulted in an even later arrest after completion of liver stage development prior to merozoite release (Haussig et al., 2011). Although, immunizations with Δ*PALM* resulted in robust long-term protection after only two immunizations in the stringent *P. berghei*-C57BL/6 model (Table 2), consistent dose-independent breakthrough infections preclude the translation to human *Plasmodium* species, unless this mutant is combined with an additional knockout that causes a similar life cycle arrest. Several attempts to target other biochemical pathways within the apicoplast, including iron-sulfur cluster biogenesis or primary reactions in heme biosynthesis, did not yield Δ*PALM*-like GAPs (Haussig et al., 2013, 2014; Rizopoulos et al., 2016).

Additional late arresting candidate genes have been identified; however, in depth analysis and immunization studies are often lacking. An interesting case is protein kinase G (PKG), which is shared across different life cycle stages and already essential during blood infection, as it plays prominent roles in merozoite egress and gametogenesis. Since generation of a Δ*PKG* parasite is incompatible with blood infection, a stage-specific knockout by FLP/FRT recombination in sporozoites was engineered to study the role(s) in pre-erythryocytic development (Falae et al., 2010). This analysis revealed a late arrest in liver stage maturation and wild type breakthrough infections due to incomplete recombination (Table 1). Immunization studies were not conclusive since challenge infections were performed only one week after high dose immunizations. However, the example of *PKG* illustrates that a tight late arrested parasite might be achieved once a suitable liver stage-specific gene at the nexus of stage conversion is identified.

## GAPs with multiple gene deletions: synergistic or antagonistic?

Development of parasite lines that harbor multiple gene deletions is likely to increase safety; however, whether synergistic or antagonistic effects modulate immunogenicity is less straightforward to predict and likely depends on the selected knockout combination.

Soon after the first proof-of-principle studies a GAP parasite line that harbors two consecutive gene deletions, namely Δ*UIS3* and Δ*UIS4*, was engineered in *P. berghei* (Jobe et al., 2007). As expected, Δ*UIS3/UIS4* parasites displayed a complete arrest in early liver stages, indicating that vaccine strains harboring multiple independent gene deletions perform safer than single knockout GAPs. Importantly, long-term protection against a high-dose sporozoite challenge infection was complete (Table 2).

In marked contrast, when a double knockout was performed for the *P. berghei* paralogs *P36p (P52)* and *P36*, which are neighboring genes and likely arose through gene duplication, safety was not improved (Annoura et al., 2012), strongly suggesting that independent genes need to be targeted in multiple gene knockout strategies. Since Δ*SLARP* GAPs lead to a complete termination of liver stage development (Aly et al., 2008; Silvie et al., 2008b), they constituted the obvious platform for combinations with knockouts of the 6-Cys gene family (Figure 1) (Mikolajczak et al., 2014; van Schaijk et al., 2014a; Kublin et al., 2017). However, it remains to be shown whether addition of *B9, P36*, and/or *P36p* (*P52*) knockout provides any additional benefit beyond perception of additional gene deletions. An important investigation with combinatorial knockouts will be the systematic expression profiling of liver stage-specific genes, as was previously done for Δ*SLARP* parasites (Silvie et al., 2008b). Such an analysis will provide first insights into the expected antigenic repertoire displayed by the respective GAPs.

Instead of adding additional gene deletions, which might not add significantly to vaccine efficacy, combination of a gene deletion with transgene expression of additional factors could amplify antigen presentation during pre-erythrocytic development. Potential avenues include perforation of the intracellular niche, i.e., the parasitophorous vacuole, activation of innate immune sensing pathways, and expression of blood stage and gametocyte antigens. Such a strategy is exemplified by the expression of perfringolysin O, a cholesterol-dependent cytolysin, in *P. berghei* Δ*PDH-E1*α parasites (Nagel et al., 2013). Addition of the transgene could substantially reduce, albeit not completely abolish, breakthrough infections of the single gene deletion, providing a rationale for further bioengineering efforts to achieve premature rupture of the PVM. However, a principal concern in gain-of-function mutants is that parasites containing mutations in the transgene promoter and other regulatory elements, which lead to a reduced transgene expression, will be swiftly selected. Therefore, GAPs that express additional *Plasmodium* antigens in order to broaden the immunogenic repertoire without further reducing parasite fitness might be a particularly rewarding research direction.

## Immune mechanisms of GAPs: the central role of effector memory CD8^+^ T cells

Cytolytic, interferon gamma (IFNγ)-secreting CD8^+^ T cells were identified early on as the key mediators of protection after radiation-attenuated sporozoite immunizations (Schofield et al., 1987; Weiss et al., 1988; Romero et al., 1989). Amongst all CD8^+^ T cell subsets, presence of those with an effector memory phenotype, i.e., CD45RB^lo^, CD44^high^, CD62L^lo^, and CD122^lo^ consistently correlated with long-lasting protection (Guebre-Xabier et al., 1999; Berenzon et al., 2003). Studies employing immunization of β_2_ microglobulin (β*2M*) knockout mice and recognition of a CSP-specific T cell clone by hepatocytes with a matching MHCI haplotype revealed that liver stage antigens are presented to the CD8^+^ T cells *via* MHCI on the surface of infected hepatocytes (White et al., 1996; Balam et al., 2012). Elimination of infected hepatocytes in immunized mice could be linked to perforin secreting cytotoxic CD8^+^ T cells and IFNγ production (Mellouk et al., 1987; Schofield et al., 1987; Malik et al., 1991; Rodrigues et al., 1993; Sano et al., 2001). Employing advanced *in vivo* imaging techniques direct proximity to antigen-specific CD8^+^ T cells was shown to be required for cytolytic killing of *Plasmodium*-infected hepatocytes (Cockburn et al., 2013; Kimura et al., 2013). Although, antigen-specific CD4^+^ T cells are expected to be central to mount an effective T cell response, their roles in B cell help to produce antibodies that inhibit sporozoite attachment and invasion of the liver is only minor. Antibodies are not essential for vaccine-induced protection, and protective CD8^+^ T cell responses can be mounted without CD4^+^ help (Schofield et al., 1987; Rodrigues et al., 1993).

Based on the insights from studies with irradiated sporozoites, CD8^+^ T cell-dependent elimination most likely is the immune effector mechanism in GAP-immunized animals. Indeed, a study employing *P. berghei* Δ*UIS3* parasites showed that immunizations of B and T cell-deficient *rag1*^−/−^ and *IFN*γ^−/−^ knockout mice failed to induce protection, confirming the central role of adaptive immune responses leading to IFNγ production in vaccine-induced immunity (Mueller et al., 2007). Immunizations of B cell-deficient mice and common laboratory mice after depletion or adoptive transfer of CD4^+^ and CD8^+^ T cells fully corroborated the central importance of CD8^+^ T cells, but neither of antibodies nor of CD4^+^ T cells. Of note, primaquine treatment results in efficient cure of liver stage parasites and reversed Δ*UIS3-*mediated protection (Mueller et al., 2007). This is in perfect agreement with the requirement for parasite persistence as metabolically active, cell cycle arrested intra-hepatic stages in order to maintain long-term protection (Scheller and Azad, 1995).

Another early study demonstrated the central role of CD8^+^ T cells, and particularly IFNγ-secreting effector memory cells, in protection induced by a *P. berghei* Δ*UIS3/UIS4* double knockout parasite line (Jobe et al., 2007). Immunization of β*2m*^−/−^ mice, which are deficient in MHCI expression and CD8^+^ T cells, abrogated vaccine efficacy. The direct comparison to RAS showed that both immunization strategies induce similar immune responses, but GAP-immunized animals displayed consistently higher levels of IFNγ-secreting effector memory cells (Jobe et al., 2007). Accordingly, it is conceivable that the insights gained from RAS immunizations can be extrapolated at least to Δ*UIS3/UIS4* GAP vaccines.

The findings obtained with *P. berghei* GAPs were fully supported by a study reporting that depletion of CD8^+^ T cells, but neither CD4^+^ T cells nor IgG1 antibodies, abolished protection by *P. yoelii* Δ*UIS3* or Δ*UIS4* parasite lines (Tarun et al., 2007). Notably, CD8^+^ T cells from *P. yoelii* GAP immunized mice induce apoptosis of infected, *in vitro* cultured hepatocytes by contact dependent, perforin-mediated cytotoxic killing, with only partial involvement of IFNγ (Trimnell et al., 2009). Short-lived CD11a^hi^, CD62L^lo^, CD44^hi^ antigen-experienced effector CD8b^+^ T cells, which also express CD11c, expanded swiftly after one immunization with *P. yoelii* Δ*UIS4* sporozoites (Cooney et al., 2013). Furthermore, these cells are KLRG1^+^CD127^−^ terminal effector cells, which upon restimulation with infected hepatocytes secrete IFN, TNFα, and IL-2 and express CD107a and perforin. Late-arresting *P. yoelii* GAPs induced larger CD8^+^ T cell responses in comparison to RAS, and the CD8α^lo^CD11a^hi^ effector memory phenotype, characterized by low expression levels of CD27, CD62L, and CD127, was increased; however, this study was done in C57BL/6 mice only (Butler et al., 2011).

While the cellular mechanisms that lead to elimination of infected hepatocytes after immunization are relatively well understood, the target epitopes displayed by infected hepatocytes remain less clear (Hafalla et al., 2011). There is growing evidence that a combination of epitopes rather than a single protective antigen correlates with vaccine-induced protection (Grüner et al., 2007; Hafalla et al., 2013). The present list of pre-erythrocytic T cell epitopes in H2-K^d^-restricted BALB/cJ and H2-K^b^-restricted C57BL/6 mice remains short (Romero et al., 1989; Hafalla et al., 2013; Murphy et al., 2013; Lau et al., 2014; Müller et al., 2017). The few immunogenic epitopes identified thus far underscore the fundamental differences of immunity elicited by eukaryotic pathogens in comparison to viruses and bacteria (Hafalla et al., 2013).

The mechanisms leading to efficient priming of effector immune responses against pre-erythrocytic parasites are still incompletely understood. Efficient priming almost certainly requires cross-presentation by dendritic cells (DCs) and is most likely a combination of DC-mediated antigen presentation in different organs, including skin-draining lymph nodes, spleen, and liver (Sano et al., 2001; Chakravarty et al., 2007; Cockburn et al., 2011; Balam et al., 2012). Most studies focused on the CSP epitope, which is abundantly expressed on the sporozoite surface and only recognized in H2-K^d^ -restricted BALB/cJ mice (Romero et al., 1989; Sano et al., 2001; Chakravarty et al., 2007; Balam et al., 2012). Whether GAPs aborting development at the trophozoite stage or later present additional antigens explaining superior CD8^+^ T cell responses remains elusive.

## Toward unified guidelines for translational GAP research

There are currently no rules established for GAP development in *P. falciparum*; however, defining a set of requirements that need to be fulfilled before moving on to *P. falciparum* studies would significantly decrease odds of clinical trial failures. An obvious criterion from murine malaria models, i.e., *P. berghei* and *P. yoelii*, is complete liver stage arrest in a large number of animals employing both murine models simultaneously. Humanized mouse models and primary human hepatocytes can provide preliminary indications on developmental arrest in the liver and, thus, potential safety concerns of a *P. falciparum* GAP vaccine candidate. However, these test systems might not provide the level of stringency necessary to predict break-through infections, which are presently best captured in pre-clinical tests in large groups of mice. In order to develop potent *P. falciparum* GAPs beyond early arresting Δ*SLARP* mutants, multiple independent gene deletions, ideally in unrelated physiological processes and in different cellular compartments, need to be considered.

Another desirable standard is robust long-term immunity against challenge infection. Identifying the correct parasite/host combination is important to draw valid conclusions from immunization experiments (Matuschewski, 2013). The *P. berghei*-C57BL/6 combination remains the most robust vaccine model to date, whereas immunizations using *P. yoelii* mutants in BALB/c mice are only of modest predictive value, since protection is easily achieved in this model. Other combinations, such as *P. berghei* and BALB/c mice, are invalid because of refractoriness of particular combinations of mouse strains and infections with certain murine malaria sporozoites (Scheller et al., 1994). This notion is illustrated in immunizations with *P. berghei* Δ*P36p* sporozoites, where only one immunization dose in an inappropriate host strain, BALB/c mice, resulted in complete protection up to 3 months after immunization (van Dijk et al., 2005). Immunization with *P. yoelii* Δ*P36p/P36* parasites also induced sterile protection in BALB/c mice after a single injection (Labaied et al., 2007); however, such protection in C57BL/6 mice was only elicited after three rounds of immunization (van Dijk et al., 2005). One important immunological difference is that protection in the *P. yoelii-*BALB/c model largely depends on the immune-dominant CD8^+^ T cell epitope of CSP. In marked contrast, T cell responses in the *P. berghei*-C57BL/6 model are likely multifactorial (Hafalla et al., 2011, 2013), which closely mimics infections in human populations with a large range of MHCI haplotypes and only infrequent CSP–specific CD8^+^ T cell responses (Offeddu et al., 2012). The recent evaluation of *Grammomys dolichurus*, an Afrotropical arboreal rodent, which naturally harbors rodent *Plasmodium* infections, as a model to study pre-erythrocytic vaccine strategies will be an important addition for preclinical evaluation of safety and immunogenicity of GAP vaccines (Conteh et al., 2017). In good agreement with the data from murine *Plasmodium* models, the natural host is highly susceptible to *P. berghei* sporozoite induced infections and multiple high immunization doses are required for robust protection (Conteh et al., 2017).

Together, comparative studies on vaccine efficacy should include the *P. berghei*-C57BL/6 model (Friesen and Matuschewski, 2011), to better define long-term protection against re-challenges. We propose that a GAP vaccine line should be evaluated for vaccine safety and immunogenicity in a two-step preclinical process before advancing to human trials. First, comparative evaluation of the candidate *P. berghei* and *P. yoelii* GAP lines in the respective mouse strains, C57BL/6 and BALB/c, will pre-select GAPs that are completely arrested upon high dose sporozoite inoculations and elicit long-lasting (>30 days) sterile immunity. Second, confirmation of safety and immunogenicity of the *P. berghei* GAP line in *G. dolichurus* immunization and challenge study provide an evidence-based rationale for translation to *P. falciparum* GAP trials.

## Roadblocks toward translation of GAPs

Intravenous injections are the preferential route of vaccine administration in mouse models; however, in real life this is inapplicable. Pediatric vaccines are exclusively administered either by intramuscular syringe injection or orally. It is evident that a malaria vaccine must adhere to the same safe routes of administration. Alternative methods that are used in drug delivery, such as intradermal, intravenous, or intraperitoneal injections, are unreasonable for a malaria vaccine to be delivered in resource-poor health infrastructures, because of the risks associated with these routes of administration, except under physician's care.

*Plasmodium* sporozoites apparently lack the ability to transmigrate through muscle or fat tissue. Accordingly, after intradermal, intramuscular, subcutaneous, or intraperitoneal injection only a very small proportion of sporozoites reach a blood vessel, resulting in reduced liver infection. This was confirmed in human volunteer studies, where intramuscular or intradermal syringe injections of cryopreserved sporozoites showed substantial delays in blood infections and high doses were required to consistently induce blood infections (Shekalaghe et al., 2014; Gómez-Pérez et al., 2015). As expected, protection after immunizations *via* these routes was negligible both in the *P. berghei* model and in volunteer studies with *P. falciparum* sporozoites (Epstein et al., 2011; Nganou-Makamdop et al., 2012). Thus, development of a GAP vaccine that can be administered in the muscle is a principal hurdle that needs to be overcome by bioengineering efforts.

The necessity for booster immunizations remains another critical limitation, which is unlikely to be removed. However, a reduction of the vaccine doses would facilitate the distribution of a malaria vaccine considerably. A better understanding of how to best amplify an initial protective CD8^+^ T cell response toward a sustained effector-memory T cell response will be essential. It is conceivable that timing of antigen expression is important, and the temporal dynamics of expansion and contraction of antigen-specific T cells need to be analyzed to inform vaccine protocols (Hafalla et al., 2013; Murphy et al., 2013; Billman et al., 2016).

Cryopreservation of sporozoites is so far the only way of efficiently preserving the infectivity of attenuated sporozoites, posing huge logistic constraints. Thus, development of a vaccine formulation, which is stable under cooled conditions or, ideally, at ambient temperature and induces long-term protection, remains a critical bioengineering milestone. *Plasmodium* sporozoites are particularly sensitive to environmental conditions, and it remains entirely speculative whether an appropriate preservation process can be implemented. A GAP vaccine formulation might be further improved by addition of an adjuvant; however, no examples exist yet for live attenuated *Plasmodium* vaccines.

## Outlook

Building on the success of live attenuated, metabolically active RAS, genetic engineering of liver stage-arrested parasites offers unprecedented opportunities to develop a precision malaria vaccine. Comparative studies with GAPs that display distinct temporal arrests during liver stage maturation provide a foundation for systems immunology approaches, which might in turn lead to a better mechanistic understanding of immune effector mechanisms that contribute to lasting protection against re-infection. Ultimately, evidence-based design of safe and effective whole sporozoite *P. falciparum* and *P. vivax* vaccines involves major research investments in preclinical murine malaria models before translation to the human parasite is warranted. Proper design of human clinical trials with predictive power for vaccine safety, negligible adverse events, and vaccine efficacy in young children living in very diverse tropical countries remains very challenging.

## Author contributions

All authors contributed equally to this work and approved the manuscript for publication.

### Conflict of interest statement

KMa is listed as inventor on international and national patents “Live genetically attenuated malaria vaccine” and “Live genetically engineered protozoan vaccine.” These patents were filed by the authors' non-profit institutions to promote the development and distribution of malaria vaccines to people in need worldwide, in accordance with a global access strategy. The other authors declare that the research was conducted in the absence of any commercial or financial relationships that could be construed as a potential conflict of interest.

## Abbreviations

β2M: β_2_ microglobulin
CAS: chemically arrested sporozoites
CSP: circumsporozoite protein
DC: dendritic cell
ETRAMP: early transcribed membrane protein
FASII: fatty acid synthesis II
G3PAT: glycerol-3-phosphate acyltransferase
G3PDH: glycerol-3-phosphate dehydrogenase
GAP: genetically arrested parasite
IK2: eukaryotic initiation factor-2α kinase
MHC: major histocompatibility complex
PALM: *Plasmodium*-specific apicoplast protein important for liver merozoite formation
PDH: pyruvate dehydrogenase
PKG: protein kinase G
PP1: eIF2α-P protein phosphatase 1
MSP1: merozoite surface protein 1
PVM: parasitophorous vacuole membrane
RAS: radiation attenuated sporozoites
SLARP/ SAP1: sporozoite and liver stage asparagine-rich protein
TRAP: thrombospondin-related anonymous protein
UIS: upregulated in infectious sporozoites.
